# Alkaline Decomposition Kinetics in Ca(OH)_2_ Medium of Mercury Jarosite

**DOI:** 10.3390/toxics14040293

**Published:** 2026-03-28

**Authors:** Sayra Ordoñez, Rubén H. Olcay, Francisco Patiño, Hernán Islas, J. Eliecer Méndez, Mizraim U. Flores, Iván A. Reyes, Miriam Estrada, Miguel Pérez

**Affiliations:** 1 Industrial Electromechanics Area, Technological University of Tulancingo, Tulancingo 43642, Mexico; eliecer@utectulancingo.edu.mx (J.E.M.); mflores@utectulancingo.edu.mx (M.U.F.); 2 Department of Metallurgical and Mining Engineering, Faculty of Engineering and Architecture, Arturo Prat University, Arturo Prat Avenue 2120, Iquique 1110939, Chile; rolcay@unap.cl; 3 Energy Engineering, Metropolitan Polytechnic University of Hidalgo, Tolcayuca 43860, Mexico; franciscopatino@hidalgo.gob.mx (F.P.); hernan.islas@hidalgo.gob.mx (H.I.); 4 Institute of Metallurgy, Autonomous University of San Luis Potosí, San Luis Potosí 78210, Mexico; alejandro.reyes@uaslp.mx; 5 SECIHTI Secretariat of Science, Humanities, Technology, and Innovation, Mexico City 03940, Mexico; 6 Academic Area of Earth Sciences and Materials, Autonomous University of Hidalgo State, Mineral de la Reforma 42183, Mexico; miguel_perez5851@uaeh.edu.mx

**Keywords:** reaction kinetics, mercury jarosite, reaction topology, decomposition, controlling stage, kinetics modeling

## Abstract

Mercury in jarosites is crucial for environmental management and metallurgy. These minerals can incorporate highly toxic heavy metals from mining waste into their structure. This study analyzes the decomposition of mercury jarosite in a Ca(OH)_2_ medium, focusing on its topological, kinetic, and modeling characteristics. Topological analysis, XRD and SEM−EDS were performed. ICP−OES was used to analyze the mercury and sulfur ions diffusing from the mercury jarosite into the Ca(OH)_2_ solution. The kinetic model that best fit the data was that of spherical particles of constant size with an unreacted core under chemical control. The XRD results did not show new crystallographic phases. SEM−EDS showed a partially decomposed particle indicating a halo and core. The experimental conditions included temperatures from 298.15 to 333.15 K, concentrations of 0.0071–0.23210 mol L^−1^ Ca(OH)_2_, particle diameters of 25–53 µm, and pH of 11.12–12.85. During the induction period, reaction orders of 1.04 and 0.44 were obtained, along with an activation energy of 77.580 kJ mol^−1^. For the progressive conversion period, the reaction orders were 0.59 and 0.15, with an activation energy of 52.124 kJ mol^−1^. The overall kinetic modeling showed favorable results, supporting the evolutionary process of the mercury jarosite decomposition reaction in an alkaline medium under different conditions. This allows prediction of when mercury could be released back into the environment in alkaline soils or lime barriers.

## 1. Introduction

Jarosite is a byproduct generated in the process of removing excess iron from the leaching solution [1] with sulfuric acid during the smelting stage of the hydrometallurgical zinc process [2]. This mineral belongs to the alunite supergroup with the general formula AFe_3_(SO_4_)_2_(OH)_6_. It is found in soil environments with acid mine drainage (AMD), in acidic rock formations, and in mining industries, where it commonly precipitates in acidic environments (pH ≤ 3) rich in sulfates [3,4]. Due to its isostructural framework, the A site can host monovalent and divalent cations (K^+^, Na^+^, NH_4_^+^, H_3_O^+^, Rb^+^, Ag^+^, Tl^+^, Ca^2+^, Sr^2+^, Ba^2+^, ½ Pb^2+^, Bi^2+^), including heavy metals such as mercury (Hg^2+^) [5,6,7].

The storage of these jarosites represents a critical environmental challenge, not only due to the loss of valuable metals [8], but also because of mercury toxicity, classified by the World Health Organization (WHO) and the Agency for Toxic Substances and Disease Registry (ATSDR) as one of the three most concerning chemicals because it causes serious health problems in humans [9,10], causing the disruption of biological functions, including neurological, kidney, and intestinal damage, cognitive impairment, and DNA (deoxyribonucleic acid) damage, increasing the risk of mutations [11,12,13].

Mercury is a volatile heavy metal whose annual atmospheric emissions, derived from both natural and anthropogenic sources, are estimated at 6000 and 11,000 tons [14,15]. In nature, it is found in the Earth’s crust with an abundance of 85 ppb and in soils with concentrations ranging from 10 and 200 ppb [16,17]. Due to its toxicity, the Environmental Protection Agency (EPA) has set a maximum limit of 2 ppb in drinking water. These regulations aim to mitigate risks to human health and the environment by strictly controlling their life cycles and prioritizing limiting discharge sources [18,19,20].

Although there are different techniques for remediating soils contaminated with mercury [21], such as solidification [22], photostabilization [23,24], phytoremediation [25,26] and chemical stabilization [27], the addition of organic and inorganic amendments [28], the application of biochar [29], and sulfur supplementation [30,31] are often effective and feasible to prevent mercury bioaccumulation in contaminated soils [32]. The study of jarosite as a retention medium in alkaline environments is limited [33]. Recent research has focused on decomposition kinetics, varying parameters such as particle size, temperature, and ion substitution in the jarosite structure using different acids, highlighting that decomposition in this medium is an alternative that reduces process costs, as jarosites are naturally acidic [33,34,35,36].

Studies have also been carried out in a basic medium, showing that reactions in sodium hydroxide are less energy-dependent than those in Ca(OH)_2_ [33,37,38,39,40]. However, the decomposition of mercury jarosite in a Ca(OH)_2_ medium has not been documented. The objective of this study is to determine the decomposition kinetics of mercury jarosite in Ca(OH)_2_, analyzing variables such as temperature, concentration, and particle size, to establish kinetic expressions and models that predict the actual kinetic process.

This research evaluates the stability of mercury jarosite under alkaline conditions similar to the acid mine drainage (AMD) neutralization scenario using lime. AMD, characterized by its high acidity and content of sulfates and heavy metals (Cu, Fe, Mn, Pb), poses a significant risk to ecosystems [41]. This environmental liability arises from the oxidation of sulfide minerals, such as pyrite and arsenopyrite, under environmental conditions [42]. To mitigate its impact, quicklime (CaO) is used, which, upon dissolving, releases hydroxide ions (OH^−^) that raise the pH and promote metal precipitation, thus optimizing effluent management [43,44,45]. Stability is determined through kinetic studies across a wide range of temperatures, Ca(OH)_2_ concentrations, and particle sizes. Reaction orders and activation energies were determined, allowing us to identify whether the process is limited by ionic diffusion or by surface chemical reaction. The development of the kinetic model will facilitate the prediction of mercury jarosite behavior.

## 2. Materials and Methods

For the topological study, a 0.5 L Ca(OH)_2_ solution of 0.1785 mol L^−1^, resulting in a pH of 12.28, was prepared by adding 0.2 g of mercury jarosite with a particle size of 38 μm. The jarosite was synthesized and characterized in a previous article [46], as described below by the addition technique: 10 mL h^−1^ of Hg(NO_3_)_2_·H_2_O 0.58 mol L^−1^ slightly acidified with concentrated hydrochloric acid was added for 10 h into a 750 mL solution of Fe_2_(SO_4_)_3_·nH_2_O 0.27 mol L^−1^ with an initial pH of 1.3, contained in a 1 L (Pyrex) glass reactor immersed in oil (Quaker State SAE 25W-60). It was placed on a heating plate with automatic temperature control (Super-Nuova/Barnstead-Thermoline, Dubuque, IA, USA) coupled to a coil condenser to recirculate water and a mechanical stirrer (IKA-RW16 basic, Staufen, Germany) with a 3-blade propeller. The solution was stirred at 400 rpm and heated to 93 °C (366 K) for 24 h. The obtained precipitate was filtered and washed with distilled water at 70 °C (343 K); it was then dried on a heating stove for 1 h at 65 °C (338 K) [46].

The Ca(OH)_2_ solution was placed in a Pyrex glass reactor, which was coupled to a heating plate with automatic temperature control set to 303.15 K, and magnetic stirring was set at 500 rpm. Here, 5 mL samples, without adding volume so as not to change the concentration of the solution, were extracted at different times. However, when applying the kinetic decomposition models, the material was balanced with the volume available at each of the sampling times. The mercury and sulfur content was analyzed by inductively coupled plasma-optical emission spectrometry (ICP-OES PerkinElmer 8300, PerkinElmer, Inc., Waltham, MA, USA). The cross section of the partially reacted solids was analyzed by scanning electron microscopy with energy-dispersive X-ray spectroscopy (SEM-EDS, JEOL JSM-6610LV instrument JEOL, Tokyo, Japan), and through X-ray diffraction (XRD Bruker D8, Billerica, MA, USA, powder diffractometer, with Ni filtered radiation from a Cu anode; kα1 (λ = 1.5406 Å); 40 kV and 35 mA; a recorded angular range of 2θ of 10–65°; step size = 0.01°; and step time = 3 s). Solids extracted from experiments conducted under the same conditions were analyzed by interrupting the reaction at 25, 50, 75, 100, 125, 160, 180, and 220 min.

Similar experiments were carried out in the kinetic study to evaluate the effect of varied concentrations at 0.0071, 0.0142, 0.0178, 0.0714, 0.1428, 0.1785, 0.2142, and 0.2321 mol L^−1^ of the Ca(OH)_2_ solution, keeping the temperature, magnetic stirring, particle size, pH and concentration constant. Finally, experiments were developed to evaluate the effect of jarosite particle size considering the particle diameter (d_0_), 25, 38, 44 and 53 µm, under constant conditions.

Analogously, the reaction progress curves were constructed according to the mass fraction of sulfur contained in the solution released from the jarosite, in contrast to time, establishing through the progressive conversion period the model that allows studying the underlying evolution of the decomposition of jarosite with respect to time, promoting the obtainment of the induction time (*t_ind_*) and the reaction rate (*k_exp_*). Here, we determine the concentration of the hydroxy ion [OH^−^], under the pH and temperature conditions of the Ca(OH)_2_ solution, considering the ionic constant of water (*k_w_*) [47]. Based on the experiments conducted, the kinetic equations of the periods comprising the decomposition process were determined.

The results obtained in the kinetic study were generated and evaluated using expressions calculated partially and globally during the induction and progressive conversion periods, taking into account the controlling reaction stage, the experimental rate constant, and the Arrhenius equation. This enabled kinetic modeling by substituting the experimental values that influence the reaction rate to validate the obtained data against the calculated data.

## 3. Results

### 3.1. Topology of the Reaction

**Figure 1:**
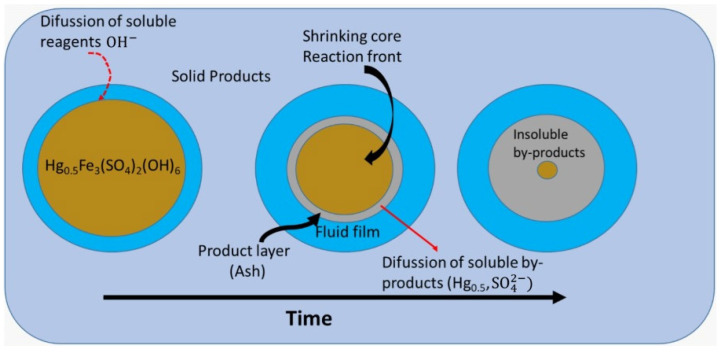
Schematic of the diffusion kinetic process of mercury jarosite decomposition.

**Figure 2:**
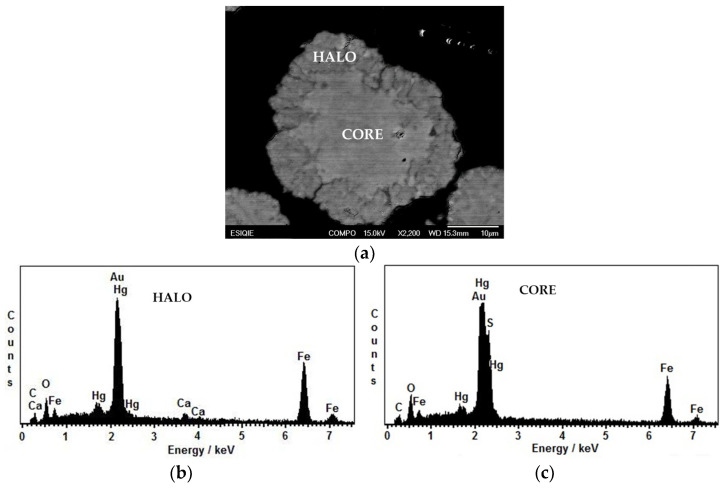
(**a**) SEM micrograph of a partially decomposed particle; (**b**) EDS spectrum of the ash halo area; (**c**) EDS spectrum of the core of the partially decomposed mercury jarosite particle.

**Figure 3:**
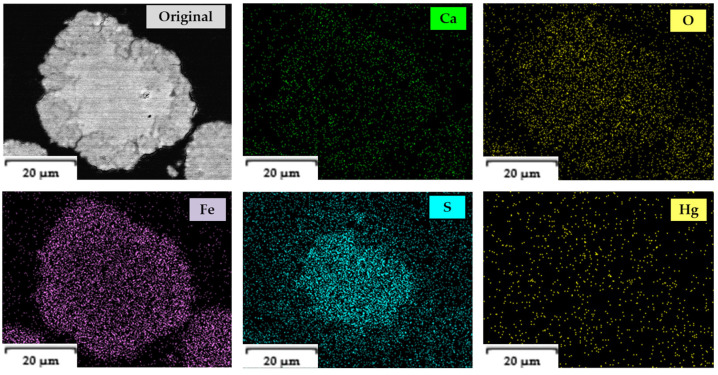
Mappings of the distribution of each element present in mercury jarosite.

**Figure 4:**
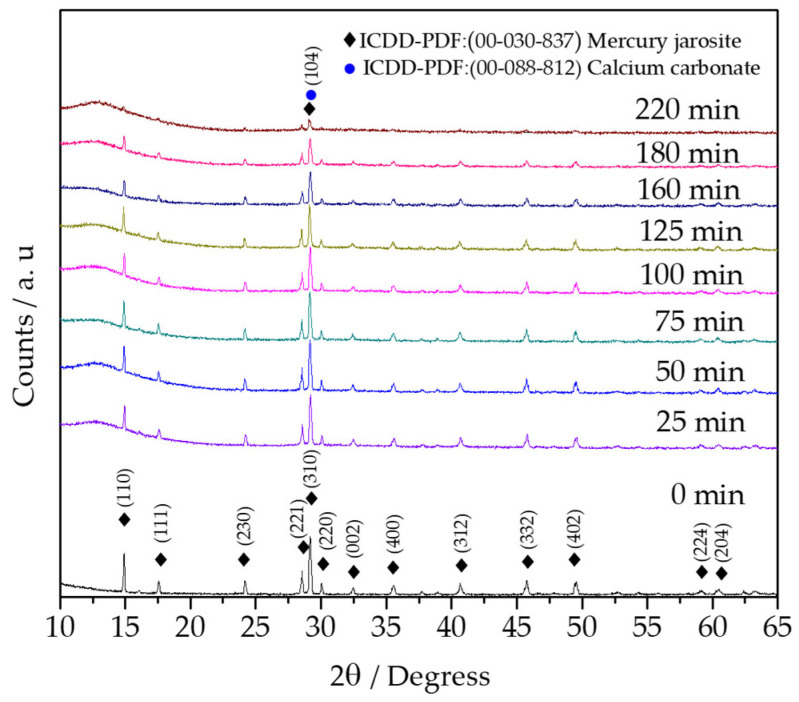
X-ray diffraction spectrum of mercury jarosite at different reaction times.

In the study of reaction topology, the concentrations of mercury and sulfur in solution were determined by ICP-OES. This analysis revealed that the reaction proceeds through distinct stages. The induction period lasts from the start of the reaction until 50 min, during which both elements remain at minimal concentrations, and no visible change is observed in the solution. This prolonged period is attributed to the formation of a small shell when the solution contacts the environment, which generates calcium carbonate (CaCO_3_) and temporarily inhibits OH^−^ ions from reacting immediately with mercury jarosite particles. The formation of calcium carbonate is due to the presence of CO_2_ in the environment, which prevents its control. As the reaction continues, a progressive conversion period (PCP) of up to 200 min is observed, evidenced by significant changes in the solution, particularly an increase in Hg and S, causing the solution to turn yellow. This color change signifies an ongoing chemical reaction [48], where sulfur and mercury ions diffuse into the solution and remain in aqueous form, while iron persists as a solid, forming amorphous iron hydroxide. These findings are supported by semi-quantitative SEM-EDS chemical analysis and X-ray diffraction (Figure 1, Figure 2, Figure 3 and Figure 4). During this stage, the reaction’s controlling mechanisms, such as transport control through ash halo diffusion (Equation (1)) and chemical control at the interface between the unreacted core and ash halo (Equation (2)), were evaluated. For both elements, X represents the mass fraction of decomposed mercury jarosite, calculated by dividing the amount of Hg or S released into the solution by the total at the end of the reaction; *k_exp_* is the experimental rate constant, and t is the time [49,50]. Correlation coefficients (R^2^) for Equation (1) were 0.9531 for S and 0.9394 for Hg; for Equation (2), they were 0.9911 for S and 0.9901 for Hg, indicating chemical control determination. The experimental rate constant (*k_exp_*) is calculated by Equation (3), where V*_m_* is the molar volume of mercury jarosite (173.78 cm^3^ mol^−1^) [51], *k_q_* is the chemical rate constant, *C_A_* is the reactant concentration, *n* is the reaction order, and *r*_0_ is the initial particle radius in μm. The reaction lasted 200–300 min, during which the concentrations of Hg and S remained constant, indicating stabilization.

1 − 3(1 − *X*)^2/3^ + 2(1 − *X*) = *k_exp_t* (1)

1 − (1 − *X*)^1/3^ = *k_exp_t* (2)

(3) $$k_{\exp}=\frac{V_{m}k_{q}C_{A}^{n}}{r_{0}}$$

According to the particle’s partially decomposed topology, the reaction proceeds over time, decreasing the core, as observed in Figure 1.

The kinetic model observed by SEM-EDS shows a halo of ashes, a reaction front, and the unaltered core, as can be visualized in Figure 2a. Obtained by EDS, the spectrum of the ash halo observed in Figure 2b is composed of Fe, Hg, Ca and O, characteristic of the alkaline medium. In Figure 2c, the spectrum of the core constituted by Fe, Hg, S, O is observed. In both spectra, signals from the epoxy resin (C) where the particles were mounted and from the Au coating used to make the sample conductive are observed.

In Figure 3, the detailed EDS distribution of each of the elements in the core and ash halo section can be observed through EDS mappings, deducing that the kinetic model that presides over the decomposition reaction is the model of spherical particles of constant size with a decreasing core. Derived from the EDS mappings, the presence of mercury and sulfur in the core occurs without reacting simultaneously as the OH^−^ ions diffuse from the solution into the particle.

In Figure 4, the diffractograms at different reaction times are observed, which reveal that as the decomposition progressed, the signals decreased, obtaining an amorphous compound, with the signal of CaCO_3_ that forms from the Ca(OH)_2_ medium identified through the International Center for Diffraction Data Power Diffraction Files database by the diffraction pattern ICDD-PDF-2 release 2022 (database 00-088-1812).

### 3.2. Kinetics of the Reaction

#### 3.2.1. Determination of the Reaction Order by Evaluating the Effect of the Concentration of the Medium

**Figure 5:**
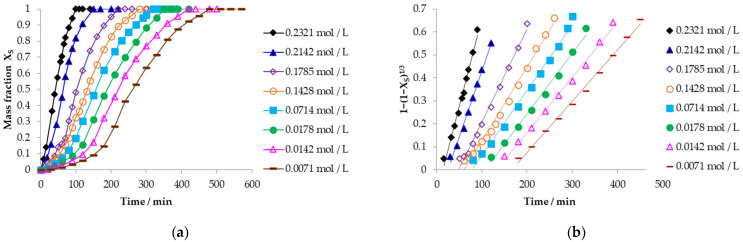
(**a**) Calculation of the reacted mass fraction during the concentration effect of Ca(OH)_2_; (**b**) application of the kinetic model to the mass fraction of the concentration effect of Ca(OH)_2_.

In relation to the study of the reaction kinetics, the analysis was carried out by measuring the mass fraction of sulfur, as a better correlation coefficient was obtained, analogous to the data the mercury element would yield. In Figure 5a, the graphs of mass fraction of sulfur for each experiment with respect to time for the concentration effect are observed, forming the decomposition curves conformed by the induction, progressive conversion, and stabilization period, concluding the reaction at 90 min when the concentration of the Ca(OH)_2_ solution is 0.2321 mol L^−1^. By decreasing the solution concentration to 0.0071 mol L^−1^, the reaction is prolonged to 450 min. Also, in Figure 5b, the graphical representation of the data for the progressive conversion period, evaluated in relation to Equation (2), is shown for the chemical control with respect to time, and it is observed that the data fit this controlling stage, with correlation coefficients ranging between 0.9901 and 0.9950. It should be noted that the progressive conversion period at a concentration of 0.0071 mol L^−1^ began at the end of the induction time (*t_ind_*), at 169.86 min, and with a higher concentration of 0.2321 mol L^−1^ it began at 13.51 min, showing that the experimental rate constant (*k_exp_*) of the reaction decreases as the concentration of the solution [52] decreases in parallel with the pH. The data for experiments conducted at different concentrations with a constant temperature and particle radius of mercury jarosite are shown in Table 1.

**Figure 6:**
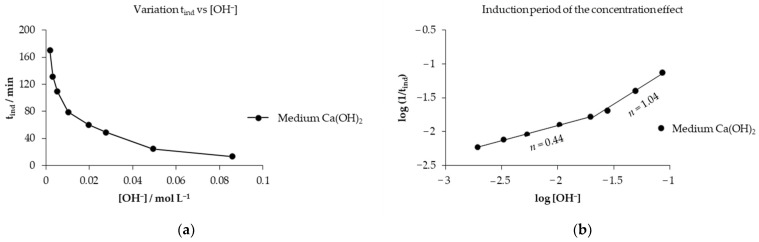
(**a**) Analysis of variation in the induction period with respect to the concentration of OH^−^; (**b**) calculation of the reaction order of the induction period.

The graph of the variation in the induction time with respect to the concentration of OH^−^ ions is visualized in Figure 6a, highlighting that the concentration of OH^−^ ions decreases, increasing the induction time, and favoring the progress of the reaction slowly. Through the concentration effect data, the reaction order (*n*) for the induction period was obtained as observed in the graph of Figure 6b on the abscissa axis, showing the log [OH^−^] with respect to the log (1/*t_ind_*) on the ordinate axis, where two reaction orders are conceived at a concentration lower than 0.0197 mol L^−1^; the reaction order is 0.44, indicating that a significant concentration gradient does not occur in the fluid film [49]. On the contrary, at a concentration greater than 0.0197 mol L^−1^, the reaction order increases to 1.04, showing that the concentration of the Ca(OH)_2_ solution will produce proportional changes in the reaction rate [49]. It is characteristic of the induction period that the reaction orders are higher because there is the formation of active sites that allow the interaction of the reaction.

**Figure 7:**
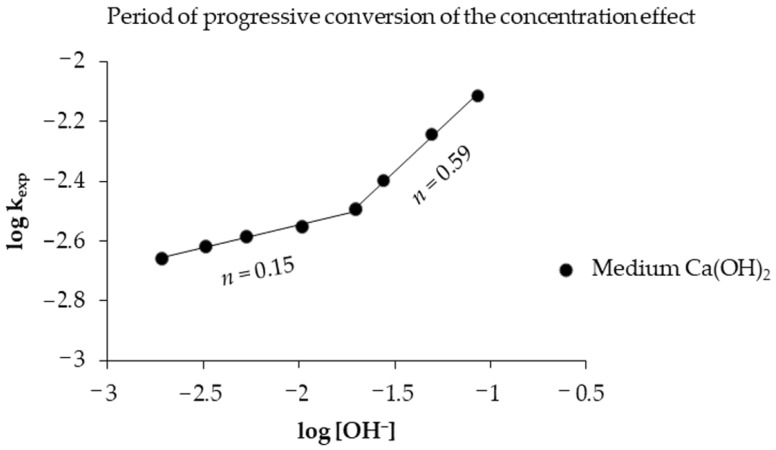
Calculation of the reaction order of the progressive conversion period of the decomposition of mercury jarosite.

Opposite to the reaction orders obtained in the progressive conversion period calculated with Equation (4) from Equation (3), which has a form of a straight line, where the slope is the reaction order, using base 10 logarithms graphically represented in Figure 7 on the abscissa axis as *logC_A_* in relation to the ordinate axis where *logk_exp_* is represented, it is observed that at a concentration greater than 0.0197 mol L^−1^ the reaction order is 0.59 and less than this concentration it is 0.15, showing that the decrease in concentration of the alkaline solution has minimal influence on the reaction rate [49] at reaction orders lower than in the induction period, allowing the reaction to develop until culminating in the stabilization period.

(4) $$\log k_{\exp}=\log\frac{V_{m}k_{q}}{r_{0}}+\mathrm{nlog}C_{A}$$

#### 3.2.2. Determination of Activation Energy by Evaluating the Effect of Medium Temperature

**Figure 8:**
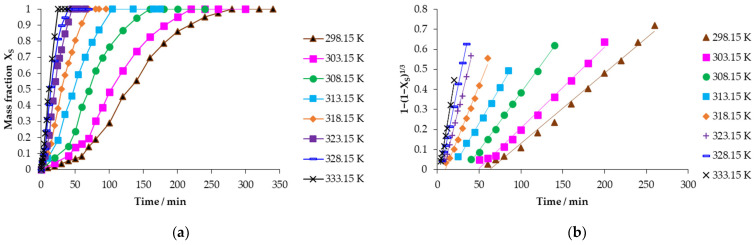
(**a**) Calculation of the reacted mass fraction during the temperature effect; (**b**) application of the kinetic model to the mass fraction of the temperature effect.

To determine the activation energy using the Arrhenius equation (Equation (5)) [53], experiments were carried out at a constant concentration and particle size with temperature varied as shown in Figure 8a. It was observed that at 298.15 K and 333.15 K, the progressive conversion period begins at the end of the induction period, at 65.16 and 2.93 min, respectively. However, it culminates at 260 and 25 min, showing that as the reaction temperature increases, the pH of the solution and the progress time of mercury jarosite decomposition decrease; simultaneously, we can observe in Figure 8b the evaluation of the data of the progressive conversion period with respect to Equation (2), with the correlation coefficient (R^2^) oscillating between 0.9900 and 0.9992, adjusting the data satisfactorily with the chemical control.

(5) $$k_{q\;}=Ae^{-E_{a}/\mathrm{RT}}$$

**Figure 9:**
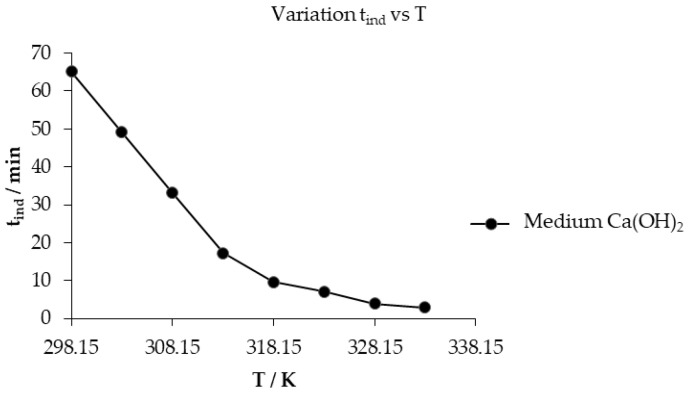
Analysis of the variation in the induction period with respect to temperature.

**Figure 10:**
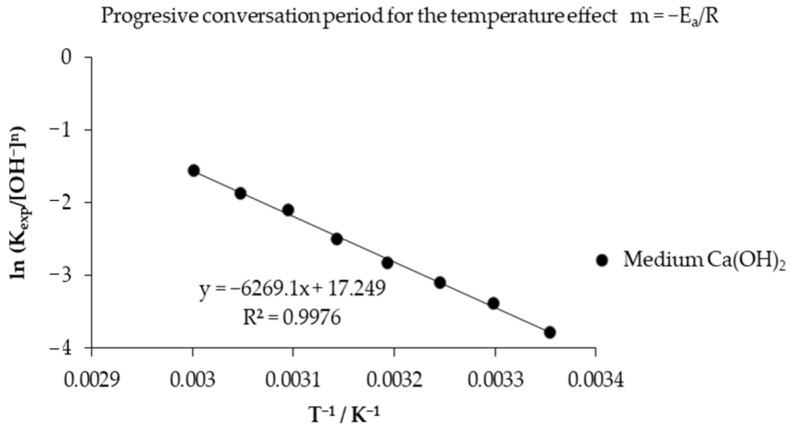
Calculation of the activation energy for the progressive conversion period.

**Figure 11:**
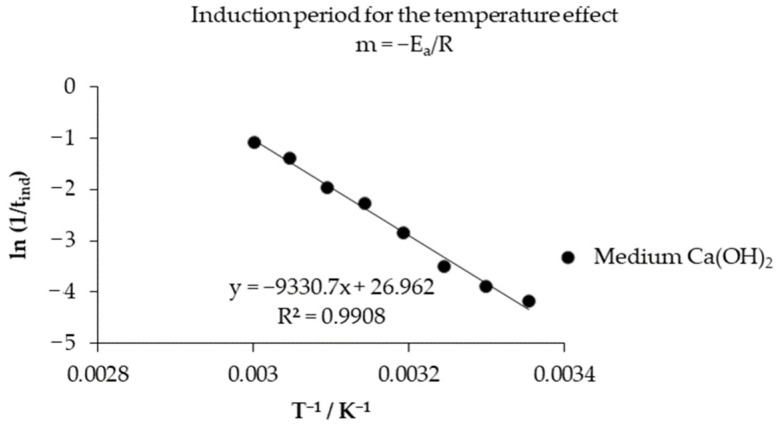
Calculation of the activation energy of the induction period.

Table 2 shows the induction times, which increase at an equidistant rate with the solution temperature, as illustrated in Figure 9. The activation energy is obtained by applying Neperian logarithms to Equation (5), where *lnk_exp_*/[OH^−^]*^n^* expresses the autonomy of the experimental rate constant (*k_exp_*) with respect to the concentration of OH^−^ ions that vary according to the temperature of the Ca(OH)_2_ solution; *lnA* is the frequency factor; *E_a_* is the activation energy; *R* is the ideal gas constant; and (1/*T*) is the inverse of the temperature [51]. Applying Equation (6), the inverse of the temperature versus *lnk_exp_*/[OH^−^]*^n^* is observed in Figure 10, for the progressive conversion period where the slope m is equal to *−E_a_*/*R*, obtaining an activation energy of 52.124 kJ mol^─1^, with a frequency factor of the antilogarithm of *lnA* = 17.249 = 3.09 × 10^7^. For the induction period, an *E_a_* of 77.580 kJ mol^─1^ was obtained with a frequency factor of the antilogarithm of *lnA* = 26.962 = 5.12 × 10^11^ calculated through the graphical representation of Figure 11, where the inverse of the temperature with respect to *ln*(1/*t_ind_*) is observed, obtaining higher values of activation energy and frequency factor compared to the progressive conversion period.

(6) $$\;\ln\frac{k_{\exp}}{{\left[{\mathrm{OH}}^{\;-}\right]}^{n}}=\mathrm{lnA}\;-\;\frac{E_{a}}{R}\left(\frac{1}{T}\right)$$

#### 3.2.3. Evaluation of the Effect of Particle Size

**Figure 12:**
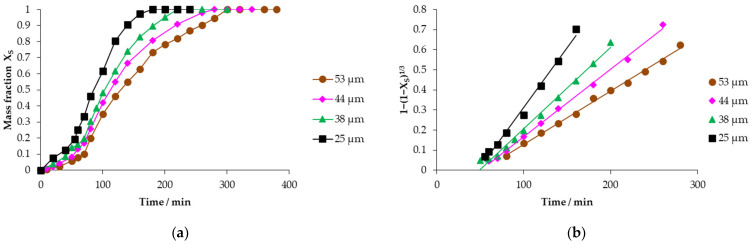
(**a**) Calculation of the reacted mass fraction during the particle size effect; (**b**) application of the kinetic model to the mass fraction of the particle size effect.

This evaluation is corroborated by the experiments carried out with a variation in the particle size of 53, 44, 38 and 25 μm, keeping constant the concentration of the Ca(OH)_2_ solution of 0.1785 mol L^−1^ and temperature 303.15 K, as observed in Figure 12a, where the decomposition curves of mercury jarosite are visualized. Figure 12b shows the data of the progressive conversion period that fit the chemical control of the reaction, obtaining correlation coefficients (R^2^) that oscillate in a range of 0.9900 to 0.9972.

**Figure 13:**
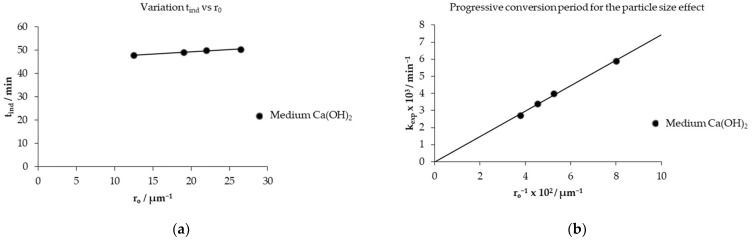
(**a**) Analysis of the variation in the induction period with respect to particle size; (**b**) calculation of the particle size effect for the progressive conversion period.

We emphasize that as the particle diameter becomes larger, the induction time is prolonged to start the progressive conversion period, as observed in Table 3. At 53 μm, the *t_ind_* is 50.48 min, ending the progressive conversion period at 280 min, and as the particle diameter decreases to 25 μm the *t_ind_* is 47.84 min, culminating the progressive conversion period at 160 min, showing that the variation in the particle size is irrelevant with respect to the induction time as visualized in Figure 13a; however, the opposite happens in the variation in the particle size with respect to the experimental rate constant, which is reciprocally proportional, as observed in Figure 13b, whose slope coexists at the origin, proving that the spherical particle model is determined by chemical control [49].

### 3.3. Kinetic Modeling

The dynamics that exist between the variables that influence the reaction rate with respect to time were studied through kinetic modeling, preceded by chemical control for spherical particles, whose mathematical model combines Equations (2), (3) and (5), which allows obtaining Equation (7), to describe the reaction according to the decomposition rate of mercury jarosite (*r_Descomposition_*) in Ca(OH)_2_ medium, considering parameters such as the reaction order, the frequency factor, the activation energy, and the *V_m_* molar volume of mercury jarosite of 173.78 cm^3^ mol^−1^ [53], *R* the ideal gas constant of 8.3145 J mol^−1^ K^−1^, and variables such as the particle size in cm, the temperature, the concentration of hydroxyls and the data shown in Table 1, Table 2 and Table 3.

(7) $$r_{\mathrm{Descomposición}\;}=\left[1\;-{\left(1\;-\;X\right)}^{1/3}\right]=\frac{V_{m}}{r_{0}}Ae^{-E_{a}/\mathrm{RT}}{\left[{\mathrm{OH}}^{-}\right]}^{n}t$$

For the induction period when the [OH^−^] is greater than 0.0197 mol L^−1^, Equation (8) is obtained; however, when the [OH^−^] is less than 0.0197 mol L^−1^, Equation (9) is generated.

(8) $$\frac{1}{t_{\mathrm{ind}}}=\frac{1}{V_{m}r_{0}}{\left[{\mathrm{OH}}^{-}\right]}^{1.04}5.12\times 10^{11}e^{-77,580/\mathrm{RT}}$$

(9) $$\frac{1}{t_{\mathrm{ind}}}=\frac{1}{V_{m}r_{0}}{\left[{\mathrm{OH}}^{-}\right]}^{0.44}5.12\times 10^{11}e^{-77,580/\mathrm{RT}}$$

**Figure 14:**
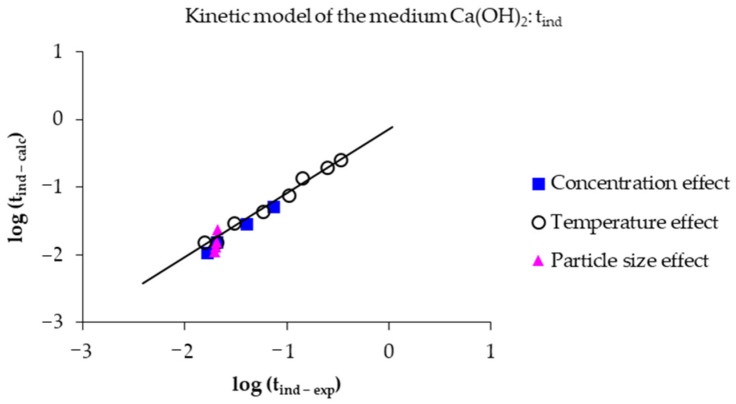
Comparative analysis of experimental data versus those calculated using Equations (8) and (9) of the induction period.

From both Equations, Figure 14 was obtained, showing the logarithm of the experimental induction time versus the logarithm of the calculated induction time. Correlation coefficient values (R^2^) of 0.9906, 0.9859, and 0.9938 were obtained for the effects of concentration, temperature, and particle size, respectively. It is noteworthy that the evaluation of the data shows similarity between the experimental and calculated values; therefore, it is considered that the experimental data are valid under these three effects.

**Figure 15:**
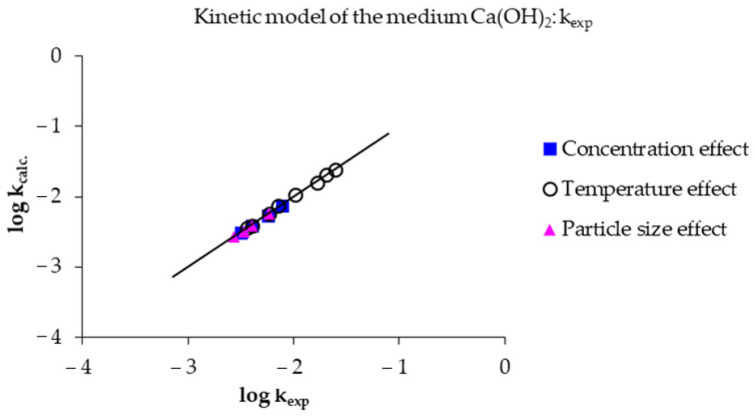
Comparative analysis of experimental data versus those calculated using Equations (10) and (11) of the progressive conversion period.

Analogously, for the progressive conversion period, the corresponding data were evaluated using Equation (10) for [OH^−^] greater than 0.0197 mol L^−1^ and Equation (11) for [OH^−^] less than 0.0197 mol L^−1^. The result is shown in Figure 15, where it is observed that the data for the logarithm of the experimental rate constant do not deviate from the data for the logarithm of the calculated rate constant. Correlation coefficients (R^2^) of 0.9987, 0.9974, and 0.9915 were obtained for the effects of concentration, temperature, and particle size, respectively.

(10) $$\;1\;-\;{\left(1-X\right)}^{1/3}=3.09\times 10^{7}e^{-52,124/\mathrm{RT}}{\left[{\mathrm{OH}}^{-}\right]}^{0.59}t$$

(11) $$\;1\;-\;{\left(1-X\right)}^{1/3}=3.09\times 10^{7}e^{-52,124/\mathrm{RT}}{\left[{\mathrm{OH}}^{-}\right]}^{0.15}t$$

**Figure 16:**
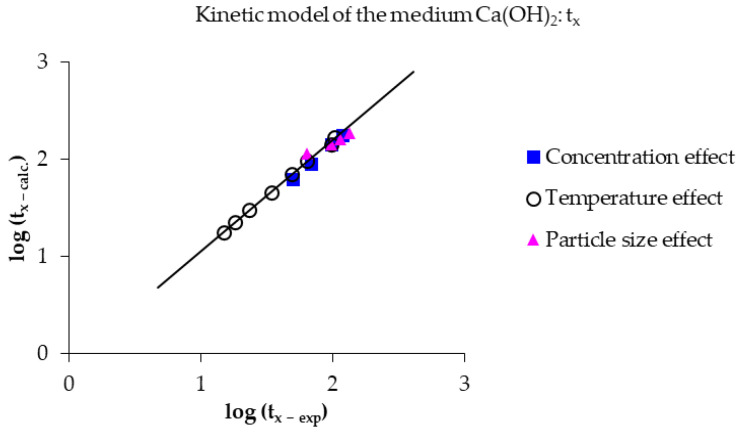
Application of the kinetic equations of the induction and progressive conversion period at a reaction time of 0.75, applying Equation (12).

Finally, to obtain a more complete and detailed view of the decomposition process of mercury jarosite in Ca(OH)_2_ medium, the global kinetic model (Equation (12)) was employed. The evolution was observed throughout the induction period, with simultaneous progressive conversion. This provides an overview of the reaction conversion process at a given time, as seen in Figure 16, which represents the logarithm of the experimental conversion time versus the logarithm of the calculated conversion time. The reaction conversion (X) was evaluated at 0.75, obtaining correlation coefficients (R^2^) of 0.9972, 0.9981, and 0.9861 for the effects of concentration, temperature, and particle size, respectively. This indicates highly favorable results for the reaction’s evolutionary process under the kinetic model.

(12) $$t_{x}=\frac{1}{\frac{1}{V_{m}r_{0}}{\left[{\mathrm{OH}}^{\;-}\right]}^{n}{5.12\times 10}^{11}e^{-\;\frac{77,580}{\mathrm{RT}}}}+\frac{1\;-{\;(1\;-\;X)}^{1/3}}{3.09\times 10^{7}e^{-\;\frac{52,124}{\mathrm{RT}}}{\left[{\mathrm{OH}}^{\;-}\right]}^{n}}$$

## 4. Discussion

Mercury occupies the cationic site in jarosite-type compounds, so under alkaline conditions it detaches from the solid and diffuses into the solution, as does sulfur. The induction period is characterized by intermolecular collisions that lead to the release of the aforementioned ions. This period is prolonged because a layer of calcium carbonate forms, preventing free collisions between the mercury jarosite and the solution. However, after the induction period, the progressive conversion period begins, during which mercury and sulfur ions diffuse freely, dissolving and remaining in aqueous form. Iron remains in the solid, forming an amorphous compound. This was confirmed by X-ray diffraction at 220 min, which revealed only crystallographic planes characteristic of calcium carbonate overlapping with the mercury jarosite remnant. Applying kinetic models of diffusion control and chemical control shows a better correlation with the latter.

Two reaction order values were obtained for the induction period. At low calcium hydroxide concentrations, the reaction order was 0.44, indicating no strong dependence on the medium; rather, the decomposition of mercury jarosite depends on other factors. For concentrations greater than 0.0197 mol L^−1^, the reaction order was 1.04, indicating a strong dependence on the reaction medium. The two reaction order values obtained during the induction period suggest a partial dependence on the reaction medium, with the 0.0197 mol L^−1^ concentration being the point at which a dependent change is observed when plotting log[OH^−^] vs. log (1/t_ind_). During the progressive conversion period, two reaction orders were also obtained, including 0.59 for concentrations greater than 0.0197 mol L^−1^, indicating that there is no total dependence on the reaction medium; therefore, other factors cause the mercury jarosite to decompose. For concentrations below 0.0197 mol L^−1^ a reaction order of 0.15 was obtained, indicating a very low dependence on the medium and suggesting that the concentration of OH^−^ ions is insufficient to decompose the mercury jarosite.

The activation energy of 77.580 kJ mol^−1^ for the induction period is due to chemical control, where more energy is required to initiate the decomposition reaction compared to the progressive conversion period, where the activation energy obtained is 52.124 kJ mol^−1^. The high *E_a_* value for the induction period reflects the formation of active sites there, which possess the capacity and energy to initiate the transition from the induction period to the progressive conversion period. Furthermore, the mercury jarosite particle is surrounded by the CaCO_3_ layer, requiring a higher activation energy for the OH^−^ ions to interact with the ash halo. Compared with similar studies, where the activation energy is $E_{a}$ = 81.715 kJ mol^−1^ for the induction period and 56.917 kJ mol^−1^ for progressive conversion [48], lower values are found in NaOH medium than in Ca(OH)_2_ medium because forming active points and allowing the reaction to propagate is more difficult [51,53]. However, both activation energies are greater than 40 kJ mol^−1^ [49], supporting, as in the study of the topology reaction and in the concentration effect, that the reaction of mercury jarosite in the Ca(OH)_2_ medium occurs by chemical control.

When mercury jarosite particles are small, their surface area increases, allowing OH^−^ ions to diffuse rapidly to the particles and decompose them in short periods. Conversely, when the particles are large, the surface area decreases, increasing the decomposition reaction time. Kinetic modeling confirmed and validated the experimentally obtained data, as correlations with the calculated data approach 1, indicating minimal error in the proposed kinetic analysis. Validating the general decomposition equation for mercury jarosite, and considering a decomposition fraction of 0.75, no significant dispersion was observed in the obtained data. This further confirms that the kinetic modeling is valid for any decomposition fraction.

## 5. Conclusions

Through the reaction topology study, a model was deduced, indicating that the reaction involves spherical particles of constant size with a decreasing core. Due to the diffusion of ions, the controlling stage was chemical control, as indicated by X-ray diffraction, which showed that no new phases formed as the reaction progressed. The concentration effect showed that as solution concentration increases, the reaction proceeds more rapidly, yielding reaction orders of 0.44 and 0.15 at concentrations below 0.0197 mol L^−1^. However, at concentrations greater than 0.0197 mol L^−1^, the reaction order is 1.04 and 0.59 for the induction and progressive conversion periods, respectively, indicating that as the concentration of the Ca(OH)_2_ solution decreases, the bioavailability of mercury in the environment minimizes, because the decomposition of mercury jarosite is slower, analogous to the decrease in temperature of the medium, in whose effect an E_a_ of 52.124 kJ mol^−1^ was determined for the progressive conversion period, as well as an E_a_ of 77.580 kJ mol^−1^ for the induction period where greater energy is required for the formation of active sites to initiate the decomposition reaction, corroborating in both periods that the reaction has chemical control because both E_a_ values are greater than 40 kJ mol^−1^, promoting the stability of the jarosite compound, making it a viable option for the retention of toxic elements, in this case mercury. This coincides the rate-controlling stage with the particle size effect and demonstrates that the experimental data are compatible with those calculated by partial expressions obtained in the kinetic modeling, which allowed predicting the real kinetic process of mercury jarosite in alkaline medium in a range of experimental conditions designated in this study.

## Figures and Tables

**Table 1:** Data on the concentration effect of Ca(OH)_2_ at a constant temperature of 303.15 K with a particle size of 38 μm of mercury jarosite.

[Ca(OH)_2_] mol L^−1^	pH	[OH^−^] mol L^−1^	*t_ind_* * min	*t_f_* ** of PCP min	R^2^ ***	*k_exp_* min^−1^	log [OH^−^]	log *k_exp_*	log(1/*t_ind_*)
0.2321	12.77	0.0859	13.51	90	0.9901	0.0077	−1.066	−2.113	−0.130
0.2142	12.53	0.0494	24.80	120	0.9930	0.0057	−1.306	−2.244	−1.394
0.1785	12.28	0.0277	49.20	200	0.9911	0.0040	−1.556	−2.397	−1.691
0.1428	12.13	0.0197	60.34	260	0.9901	0.0032	−1.706	−2.494	−1.780
0.0714	11.85	0.0103	78.92	300	0.9912	0.0028	−1.986	−2.552	−1.897
0.0178	11.56	0.0053	109.26	330	0.9913	0.0026	−2.276	−2.585	−2.038
0.0142	11.35	0.0033	131.45	390	0.9944	0.0024	−2.486	−2.619	−2.118
0.0071	11.12	0.0019	169.86	450	0.9950	0.0022	−2.716	−2.657	−2.230

*t_ind_* * = induction time, *t_f_* ** = end time of the progressive conversion period, R^2^ *** = correlation coefficient.

**Table 2:** Data on the temperature effect of Ca(OH)_2_ at a constant concentration of 0.1785 mol L^−1^ and particle size of 38 μm for mercury jarosite.

Temperature K	pH	[OH^−^] mol L^−1^	*t_ind_* * min	t*_f_* ** of PCP min	R^2^ ***	−*log k_w_*	*k_exp_* min^−1^	1/T K^−1^	In(*k_exp_/*[OH^−^]*^n^*)	ln(1/*t_ind_*)
298.15	12.65	0.0451	65.16	260	0.9935	13.995	0.0036	0.00335	−3.7794	−4.1769
303.15	12.28	0.0277	49.20	200	0.9911	13.836	0.0040	0.00329	−3.3843	−3.8958
308.15	12.19	0.0319	33.08	140	0.9983	13.685	0.0058	0.00324	−3.0965	−3.4991
313.15	12.00	0.0287	17.22	85	0.9958	13.542	0.0071	0.00319	−2.8297	−2.8463
318.15	11.90	0.0312	9.60	60	0.9900	13.405	0.0104	0.00314	−2.4988	−2.2623
323.15	11.83	0.0358	7.12	40	0.9947	13.275	0.0168	0.00309	−2.1016	−1.9636
328.15	11.67	0.0329	3.98	35	0.9992	13.152	0.0203	0.00304	−1.8616	−1.3813
333.15	11.48	0.0279	2.93	20	0.9913	13.034	0.0250	0.00300	−1.5544	−1.0770

*t_ind_* * = induction time, t*_f_* ** = end time of the progressive conversion period, R^2^ *** = correlation coefficient.

**Table 3:** Data of the particle size effect in Ca(OH)_2_ medium at a constant concentration of 0.1785 mol L^−1^ and temperature 303.15 K.

*d*_0_ * µm	*r*_0_ ** µm	pH	*t_ind_* *** min	t*_f_* **** of PCP min	R^2^ *****	*k_exp_* min^−1^	*k_exp_* × 10^3^ min^−1^	*r*_0_^−1^ × 10^2^ µm
53	26.5	12.33	50.48	280	0.9966	0.0027	2.7	3.7735
44	22.0	12.29	50.02	260	0.9972	0.0034	3.4	4.5454
38	19.0	12.28	49.20	200	0.9911	0.0040	4.0	5.2631
25	12.5	12.85	47.84	160	0.9900	0.0059	5.9	8.0000

*d*_0_ * = initial diameter of the particle, *r*_0_ ** *=* initial radius of the particle, *t_ind_* *** = induction time, t*_f_* **** = end time of the progressive conversion period, R^2^ ***** = correlation coefficient.

## Data Availability

The original contributions presented in this study are included in this article. Further inquiries can be directed to the corresponding author.

## Abbreviations

The following abbreviations are used in this manuscript:

AMD	Acid mine drainage
WHO	World Health Organization
ATSDR	Agency for Toxic Substances and Disease Registry
DNA	Deoxyribonucleic acid
EPA	Environmental Protection Agency
ICP-OES	Inductively coupled plasma–optical emission spectrometry
SEM-EDS	Scanning Electron Microscopy with Energy Dispersive X-ray Microanalysis
XRD	X-ray Diffraction
PCP	Progressive Conversion Period
ICDD-PDF	International Center for Diffraction Data Power Diffraction Files database by the diffraction pattern
